# RSV and HMPV Infections in 3D Tissue Cultures: Mechanisms Involved in Virus-Host and Virus-Virus Interactions

**DOI:** 10.3390/v13010139

**Published:** 2021-01-19

**Authors:** Johan Geiser, Guy Boivin, Song Huang, Samuel Constant, Laurent Kaiser, Caroline Tapparel, Manel Essaidi-Laziosi

**Affiliations:** 1 Department of Microbiology and Molecular Medicine, Faculty of Medicine, University of Geneva, 1211 Geneva, Switzerland; Johan.Geiser@unige.ch (J.G.); Laurent.Kaiser@hcuge.ch (L.K.); caroline.tapparel@unige.ch (C.T.); 2 Research Center in Infectious Diseases, CHU of Quebec and Laval University, Quebec City, QC 47762, Canada; guy.boivin@crchudequebec.ulaval.ca; 3 Epithelix Sàrl, 1228 Geneva, Switzerland; song.huang@epithelix.com (S.H.); samuel.constant@epithelix.com (S.C.); 4 Division of Infectious Diseases, Geneva University Hospital, 1211 Geneva, Switzerland

**Keywords:** HMPV, RSV, airway epithelia, single and dual infections, innate immunity and IFN response

## Abstract

Respiratory viral infections constitute a global public health concern. Among prevalent respiratory viruses, two pneumoviruses can be life-threatening in high-risk populations. In young children, they constitute the first cause of hospitalization due to severe lower respiratory tract diseases. A better understanding of their pathogenesis is still needed as there are no approved efficient anti-viral nor vaccine against pneumoviruses. We studied Respiratory Syncytial virus (RSV) and human Metapneumovirus (HMPV) in single and dual infections in three-dimensional cultures, a highly relevant model to study viral respiratory infections of the airway epithelium. Our investigation showed that HMPV is less pathogenic than RSV in this model. Compared to RSV, HMPV replicated less efficiently, induced a lower immune response, did not block cilia beating, and was more sensitive to IFNs. In dual infections, RSV-infected epithelia were less permissive to HMPV. By neutralizing IFNs in co-infection assays, we partially prevented HMPV inhibition by RSV and significantly increased the number of co-infected cells in the tissue. This suggests that interference in dual infection would be at least partly mediated by the host immune response. In summary, this work provides new insight regarding virus-host and virus-virus interactions of pneumoviruses in the airway epithelium. This could be helpful for the proper handling of at-risk patients.

## 1. Introduction

Respiratory infections constitute the second cause, after prematurity, of death in children under the age of 5 years [1]. In particular, Respiratory Syncytial Virus (RSV) and human Metapneumovirus (HMPV) are the most frequent etiological agents of acute lower respiratory tract infections, namely bronchiolitis and pneumonia, and are responsible of about 50% of the hospitalization cases (around 40% for RSV and 10% for HMPV) in the pediatric population [2,3]. To date, there is no approved antiviral or vaccine against these two viruses (except ribavirin for RSV that is not highly effective). This might be a consequence of the poor understanding of their pathogenicity [4,5]. RSV and HMPV belong to the *Pneumoviridae* family according the new classification of the *Mononegavirales* order in 2016 [6]. In addition to their similar illness manifestation, the two viruses share epidemiological features like their co-circulation worldwide during winter and spring [4,5]. A number of epidemiological studies have recently reported frequent cases of RSV–HMPV co-detection in patients with controversial conclusions concerning the type of association between the two viruses [7,8,9]. Further investigation is still needed in order to elucidate the mechanism of RSV–HMPV interaction leading to disease attenuation or exacerbation.

Discovered not long ago [10,11], HMPV has been extensively studied in in vitro and in vivo in mouse models during the two last decades [4,5,11,12,13]. In addition to these models, a number of studies in 3-dimensional (3D) in vitro reconstituted tissues have been conducted lately for a better understanding of RSV infections ex vivo [12,14,15,16,17,18]. In two recent works and using a similar ex vivo model, we have recently shown the moderate pathogenicity of RSV compared to influenza H3N2 virus (highly pathogenic) and coronavirus HCoV-OC43 (with low pathogenicity) [14] and its capacity to block rhinovirus (RV) replication in dual infections by triggering the interferon (IFN) response [15]. In the present study, the pathogeneses of RSV and HMPV viruses were compared in single and dual infections using differentiated 3D cultures. We showed that HMPV is less pathogenic than RSV; first based on their replication kinetics and capacity to induce host response in single infection; and second based on RSV ability to interfere with HMPV replication in dual infections.

Our investigations using ex vivo and in vitro models also suggested that their pathogenicity in single and dual infections would rely on their differential sensitivity to the host innate immunity response against viral infections. By comparing RSV and HMPV infections, we provide new answers regarding the mechanisms of virus-host and virus-virus interactions of these two pneumoviruses. It also highlights the relevance of the ex vivo model using 3D airway epithelial tissues to study respiratory viruses compared to the 2D in vitro model.

## 2. Materials and Methods

### 2.1. Viruses

The recombinant strains, HMPV group A strain C-85473 [13] and RSV-Amcherry [19], were used in this study. They encoded GFP (green fluorescent protein) and mcherry (red protein), respectively. Viral stocks were produced in LLC-MK2 (Rhesus Monkey Kidney Epithelial Cells) cells at 37 °C under a 5% of CO_2_ atmosphere in OptiMEM (Life technologies, Waltham, MA, USA) supplemented with 0.0002% trypsin (Sigma Aldrich, St. Louis, MO, USA). To prevent the loss of infectivity during the storage at −80 °C, viral stocks were diluted in a cryo-conservation solution (final concentration HEPES 0.05 M, MgSO_4_ 0.1 M, pH 7.5). They were finally titrated in LLC-MK2 cells.

### 2.2. Infection in Ex Vivo Model

Ex vivo infections were performed in a commercially available in vitro reconstituted 3D airway epithelium called MucilAir^TM^ produced by Epithelix SARL, Geneva, Switzerland [www.epithelix.com]. These tissues were cultured in an air–liquid interface (ALI) system at 37 °C under a 5% of CO_2_ atmosphere. The basolateral medium was changed daily during the infection. Single and dual infections, with multiplicity of infection (MOI) around 0.02, were performed as previously described [14,15]. During single and dual infections, apical washes and the basolateral media were collected daily. In order to test the involvement of IFN in RSV and HMPV infections, the same single and dual infection assays were then also performed in the presence or absence of antibodies specifically neutralizing type I IFN, a mixture of type I IFN antibody diluted 1/50 (39000-1, pbl assays science, Piscataway, NJ, USA) and type III IFN, 10 μg/mL of anti-IFN-λ1 (MAB15981-100, RandD, Minneapolis, MN, USA), which were added daily to the basolateral medium.

### 2.3. Infection in In Vitro Model

In vitro infections were performed in A549-derived cells (A549: Adenocarcinomic human alveolar basal epithelial cells), namely IFNLR1 (also called IL-28RA) or STAT1 knock out (KO) cells, including positive control (CTRL). These cells were a gift from the research group of Pr John McLauchlan (MRC- University of Glasgow Centre for Virus Research, Glasgow, United Kingdom) generated using CRISPR -Cas9 ‘nickase’ DNA plasmid co-transfection (manuscript in preparation). RSV and HMPV infections (MOI around 0.04) were performed in these A549-derived cells at 37 °C under a 5% of CO_2_ atmosphere in OptiMEM supplemented with 0.0002% trypsin.

### 2.4. Pretreatment Assays

For type I and type III pretreatments, HMPV- and RSV-infected tissues were continuously exposed to IFN treatment starting from day −1 (24 h prior infection) to 3 days post-infection. For each tissue, 1000 IU of IFN-α2a (Roferon A, Roche, Basel, Switzerland) or 5 ng/mL of IL-29/IFN-λ1 (RandD system, Minneapolis, MN, USA, 1598-IL-025) were added to the basolateral medium, which was changed daily during the infection.

### 2.5. Quantitative Real Time RT-PCR (RT-qPCR)

Virus RNA was extracted using E.Z.N.A viral RNA extraction kit (Omega, Norcross, USA, R6874-02) and quantified by real-time RT-qPCR using a specific set of primers and probes targeting RSV-A [20] and HMPV (primers forward 5′-CATAYAARCATGCTATATTAAAAGAGTCTCA-3′ and reverse 5′-CCTATYTCWGCAGCATATTTGTARTCAG-3′; and probe 5′-Fam-CAACHGCAGTRACACCYTCATCATTRCA-TAMRA-3′) and QuantiTect probe RT-qPCR kit (Qiagen, Amtsgericht Düsseldorf, The Netherlands, 204443) in a StepOne Applied Biosystems thermocycler. Results were analyzed using the StepOne^TM^ V2.0 Software (Applied Biosystems, Waltham, MA, USA).

### 2.6. Immunofluorescence (IF)

Five days post-infection (dpi), HMPV, and RSV-infected tissues were permeabilized with 1% Triton (Fluka, Charlotte, NC, USA) for 15 min at RT. Zonula occludens-1 (ZO-I), a marker of tight junctions, was stained at 4 °C overnight with antibody targeting ZO-I (Invitrogen, Karlsbad, CA, USA, 33-9100) diluted 200X in PBS containing 5% BSA and 0.2% triton and then with secondary antibodies alexafluor anti mouse 647 (Invitrogen, A31571) diluted 1/1000 in PBS. DAPI (4’, 6-diamino-2-phenylindole) was used to stain the cell nuclei. Three PBS washes were performed between each step. Finally, pictures were acquired using Zeiss LSM 700 Meta confocal microscope with a 63.6/1.4 objective. The same protocol was performed in infected A549-derived cells but without ZO-I staining. For quantification, images of stained cells (minimum 5 per sample) acquired using confocal microscopy (from infected tissues) or imageXpress (from infected A549 cells) were processed and infected cells (red for RSV and green for HMPV) were scored using MetaMorph V7.10 or MetaXpress V5.1 softwares, respectively.

### 2.7. Quantification of Cytokine Induction

Interferon lambda (IFN-λ1/λ3, IL-29/IL-28B), CXC motif chemokine 10 (IP-10), Interleukin-8 (IL-8), interleukin-6 (IL-6), and C-C motif ligand 5 (RANTES) were measured using purchased ELISA (enzyme-linked immunosorbent assay) kits (R&D, Minneapolis, MN, USA, DY1598B-05, DY266-05, DY208-05, DY206-05, and DY278-05, respectively) according to the manufacturer’s instructions.

### 2.8. Trans-Epithelial Electrical Resistance (TEER)

On the last day of infection, TEER was measured for each tissue with an EVOM volt ohmmeter (World Precision Instruments, Sarasota, FL, USA) as previously described [14].

### 2.9. Statistical Analyzes

Values were expressed as means ± SEMs with a minimum of 3 biological replicates. Statistical significance was calculated using two-way ANOVA or the unpaired *t*-test (biological replicates ≥ 3). *** *p* < 0.001; ** *p* < 0.01 and * *p* < 0.05).

## 3. Results

### 3.1. RSV Seems More Pathogenic than HMPV Regarding Viral Replication and the Induction of Host Response

**Figure 1:**
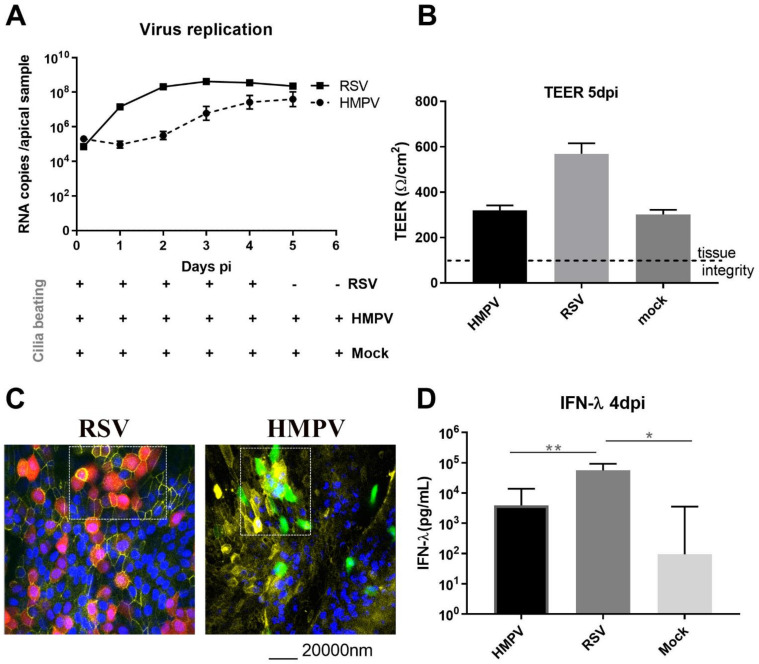
Differential pathogenesis of HMPV and RSV in in vitro reconstituted airway epithelia. Epithelial tissues were inoculated apically with RSV and HMPV (MOI = 0.02) and washed three times after 4H. (**A**) Viral RNA loads were then measured by RT-qPCR from daily collected apical washings. Cilia beating was determined (cf. movies in Supplemental Data). +: cilia are still beating; −: cilia movement is blocked by the infection. (**B**) In the same infected tissues, TEER was measured at day 5 pi. (**C**) Images acquired by confocal microscopy from the apical surface of the RSVmcherry (red cells, left panel) and HMPV/GFP (green cells, right panel) infected tissues after fixation and staining of tight junction marker, ZO-I, (yellow, a pseudo-color used instead of the original one -magenta- for a better interpretation of the image) and nuclei (blue) at 5 dpi (of note, infected cells were only detected at the apical surface as shown in Figure S1). White squares highlight groups of infected cells without syncytia formation. (**D**) IFN-λ induction was also measured from basal samples at 4 dpi by ELISA in mock, RSV, and HMPV-infected tissues. Statistical significance relative to non-infected tissues was calculated using the two-way ANOVA, N = 4. ** *p* < 0.01; * *p* < 0.05.

**Figure 2:**
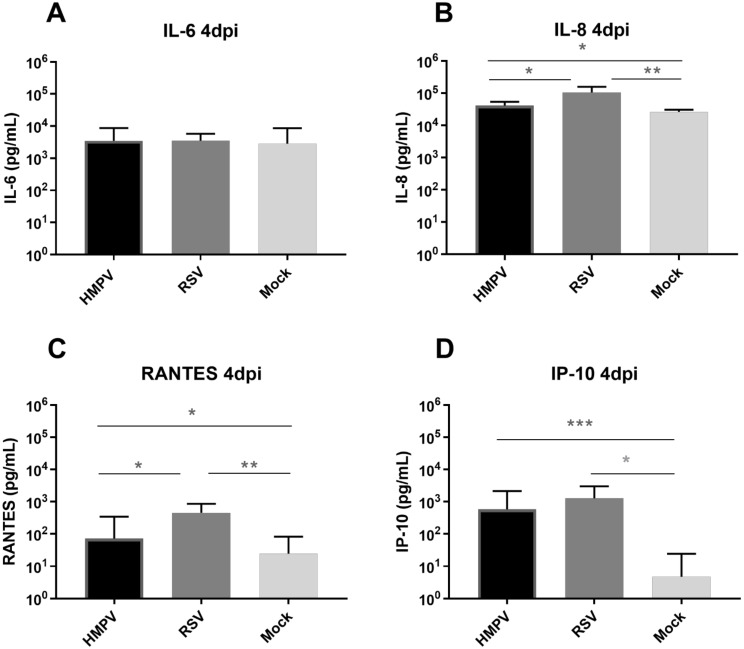
Cytokine induction by RSV and HMPV in in vitro reconstituted airway epithelia. IL-6 (**A**), IL-8 (**B**), RANTES (**C**), and IP-10 (**D**), levels were measured 96H post-infection with RSV or HMPV in daily collected basal medium using ELISA. Statistical significance was calculated using the two-way ANOVA, N = 4. *** *p* < 0.001;** *p* < 0.01; * *p* < 0.05.

**Figure 3:**
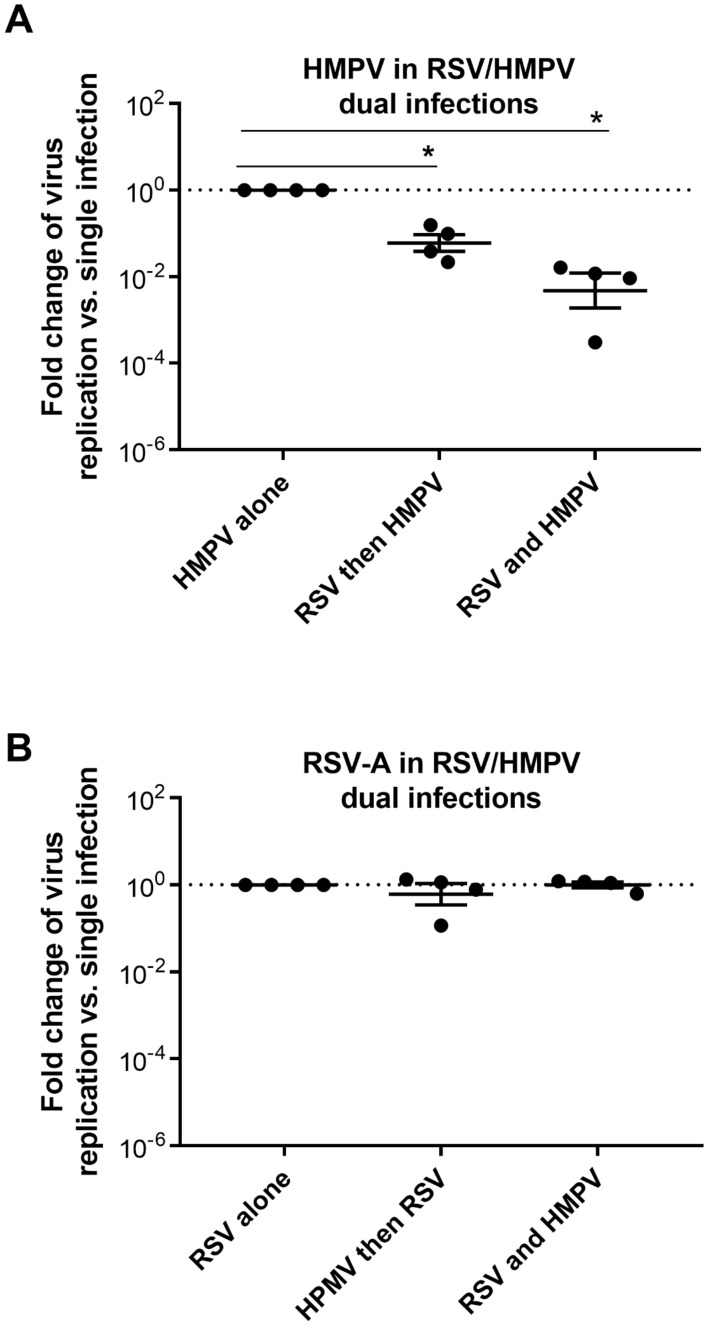
RSV–HMPV dual infections in in vitro reconstituted tissues. HMPV–RSV dual infections were performed in MucilAir^TM^. Apical replications of HMPV (**A**) and RSV (**B**) in sequential (with an interval of 2 days) and co-infections versus single infections were quantified 5 dpi by RT-qPCR. Fold changes in viral replication compared to a single infection are represented in decimal logarithm. Statistical significance calculated using the two-way ANOVA, N = 4, was shown (* *p* < 0.05).

In order to compare ex vivo infections by pneumoviruses, Mucilair^TM^ tissues were inoculated with RSV and HMPV at an MOI of around 0.02. Kinetics of viral replication was quantified by RT-qPCR from daily apical tissue washings. As expected [14], RSV replication peaked at 2–3 dpi with around 10E8 viral RNA copies. Infection blocked cilia beating from day four (Figure 1A,D). Conversely, in HMPV infection, the peak of replication was delayed by at least two days, and the cilia beating was not affected (lower panel of Figure 1A and movies in Supplementary Data: Videos S1–S3). None of these viruses affected tissue integrity (Figure 1B). Immuno-staining images suggested that RSV and HMPV infections were contained at the apical surface of the airway epithelia tissues (apical section in Figure 1C and Figure 3D reconstitution of the tissue in Figure S1) but without evidence of syncytia formation in the tissue even in areas where cells were grouped (Figure 1C). RSV showed significantly higher induction of type III interferon (IFN) compared to HMPV (>1 log higher) and non-infected tissues (2–4 log higher), while this IFN-λ was not significantly induced by HMPV compared to non-infected tissues (Figure 1D). Both viruses also significantly induced IL-8 (10E4 to 10E5 pg/mL), IP-10 (around 10E3 pg/mL), and RANTES (10E2 to 10E3 pg/mL) but not IL-6 (Figure 2). The level of induction of these three pro-inflammatory cytokines was higher (0.5–1 log) upon RSV infection compared to HMPV. In conclusion, the comparison of RSV and HMPV infections in reconstituted airway epithelia showed that, despite some similarities regarding their tropism and their effect on tissue integrity, HMPV appeared less pathogenic than RSV considering virus replication, cilia beating inhibition and induction of the immune response, namely IFN-λ and other cytokines.

### 3.2. HMPV Replication Is Decreased in the Presence of RSV

In order to assess HMPV–RSV interaction in dual infections, sequential and co-infections were performed as previously described [15] by inoculating these viruses at the same MOI (0.02) in the same tissue with an interval of 2 days or at the same time, respectively. The effect on viral replication in sequential and co-infections was determined at 5 dpi and compared to viral replication in a single infection. Figure 3A shows that RSV-infected tissues were less permissive to HMPV. HMPV replication decreased more significantly in the case of co-infection (more than 2log decrease) compared to sequential infection (around 1log); while comparable levels of RSV replication were observed in the presence and absence of HMPV (Figure 3B). Altogether, dual infections assays showed that HMPV replication was blocked by prior RSV infection, whereas RSV was not affected by the presence of HMPV.

### 3.3. HMPV Is More Sensitive to Type I and III IFN Pretreatments than RSV

**Figure 4:**
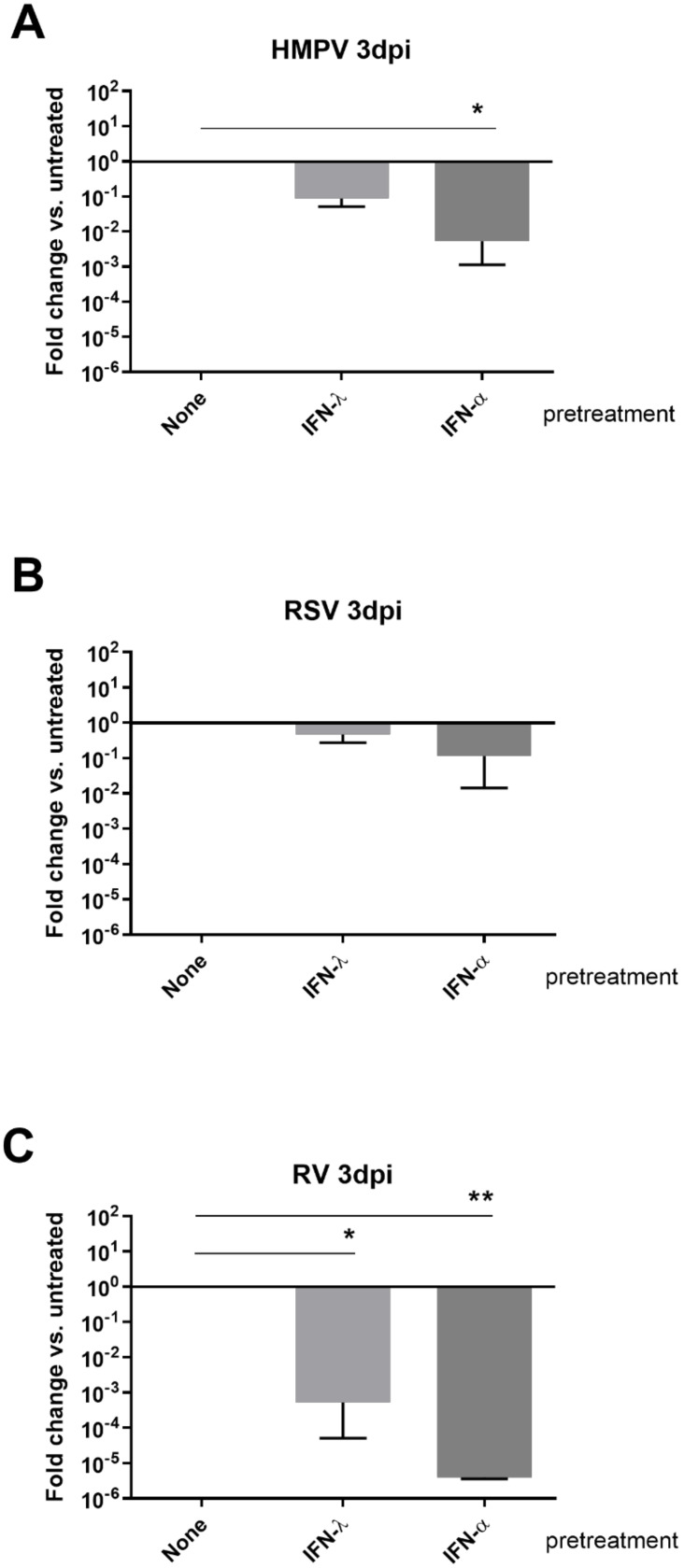
Differential sensitivity of pneumoviruses to type I and type III IFN treatments. Epithelial tissues were treated with 5 ng/mL IFN-λ (type III) or 1000IU IFN-α (type I) and infected 24 h later with HMPV (**A**), RSV (**B**), or RV (**C**). Tissues were then continuously exposed to IFNs for 3 days as described in the material and methods. Viral replication was quantified by RT-qPCR from apical samples collected at 3 dpi from epithelial tissues. Fold changes in viral replication relative to untreated tissues are represented in decimal logarithm. Statistical significance calculated using two-way ANOVA, N = 3, was shown (** *p* < 0.01 and * *p* < 0.05).

To further investigate the sensitivity of these two pneumoviruses to interferons, epithelial tissues were treated with 5 ng/mL IFN-λ (type III) or 1000IU IFN-α (type I) from day −1 (24H prior infection) until day 3 pi as previously described [15] and in details in the material and methods. Viral replication was then quantified at 3 dpi and compared in IFN pretreated versus untreated tissues (Figure 4A,B). As shown in Figure 4A, IFN-α had the strongest anti-viral effect on RSV and HMPV replication (around 1log and more than 2log fold decrease, respectively). Although less efficient than IFN-α, a slight but not significant decrease of HMPV (10-fold) was observed upon IFN-λ pre- and continuous-treatment, while no inhibition was observed for RSV. The effect of type I and III IFN pretreatments was always higher (around 10-fold) on HMPV in comparison to RSV. The effect of IFNs on pneumoviruses was globally lower than that of RV, used as a positive control [15] in these experiments (Figure 4C).

**Figure 5:**
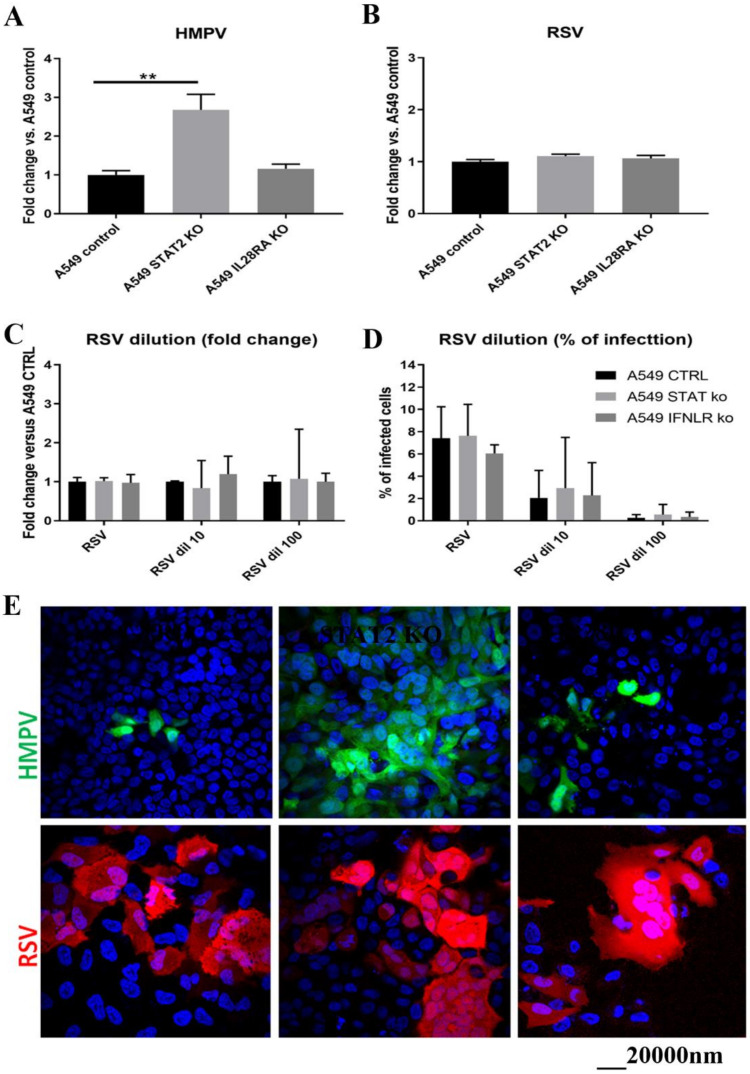
HMPV but not RSV replication increased in A549 cells in the absence of STAT1. A549 CTRL, STAT1 KO, and IL-28RA KO were infected by RSV and HMPV (MOI around 0.04). The percentage of HMPV (**A**) and RSV (**B**) infected cells was represented relative to A549 CTRL. RSV replication was also determined from A549-derived cells infected with RSV as in A and B and diluted 10 and 100 folds were represented in fold change relative to A549 CTRL from each inoculum concentration (**C**) and in the percentage of infected cells (**D**). (**E**): Confocal microscopy images of A549-derived cells infected with HMPV (green, upper panel) and RSV (red, lower panel). Nuclei were stained by DAPI (blue). Statistical significance calculated using two-way ANOVA, N = 4, was shown (** *p* < 0.01).

The sensitivity of HMPV and RSV to IFN-λ and IFN-α was also assessed using STAT1 or IL-28RA A549-knock-out (KO) cells where downstream, respectively, type I and type III or type III only IFN pathway were blocked (Figure 5). Interestingly, HMPV replication was significantly enhanced in the absence of STAT1. A slight and non-significant increase of HMPV replication was also observed in the absence of IL-28RA (Figure 5A,E). In contrast, RSV replication was not altered by the absence of IFN induction (Figure 5B,E), even with lower MOI (Figure 5C,D). As in the pretreatment assays, these data confirmed that HMPV, in contrast to RSV, was sensitive to type I but likely also to type III IFNs to a lower extent. Of note, the absence of downstream IFN pathway induction in STAT1 KO and IL-28RA KO cells was checked in these experiments (Figure S2). In summary, HMPV showed higher sensitivity to IFNs than RSV.

### 3.4. HMPV–RSV Interactions Involve IFN Response

**Figure 6:**
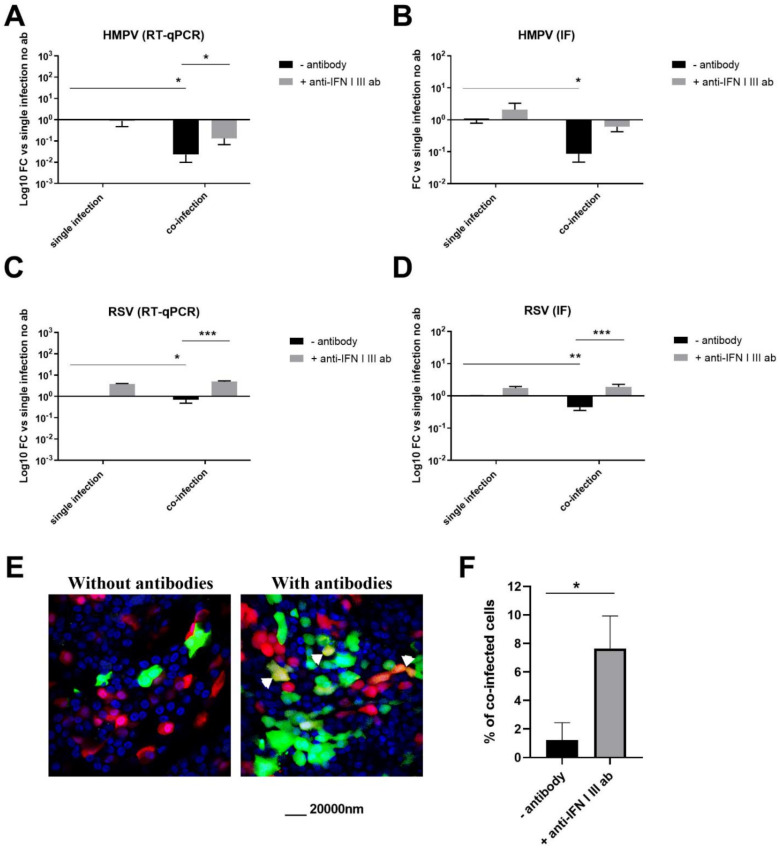
RSV–HMPV interaction would involve IFN pathways. HMPV–RSV single and co-infections were performed in 3D cultures as described in Figure 4 in the presence or absence of neutralizing antibodies against type I and III IFNs. Replication of HMPV (**A**,**B**) and RSV (**C**,**D**) versus single infections were quantified 5 dpi by real-time RT-PCR (**A**,**C**) or by MetaXpress from IF images (**B**,**D**). Fold changes in viral replication compared to a single infection without antibodies are represented in decimal logarithm. Statistical significance was calculated using two-way ANOVA, N = 3, and shown (*** *p* < 0.001; ** *p* < 0.01 and * *p* < 0.05). (**E**) From the same assays, an example of confocal microscopy images of tissues co-infected by HMPV (green) and RSV (red), in the presence (right panel) or absence (left panel) of IFN neutralizing antibodies, used for quantification. Yellow cells showing co-infected by both viruses indicated by white arrows. Nuclei were stained by DAPI (blue). (**F**) Percentage of co-infected cells relative to total infected cells, in the presence or absence of anti-type I and III IFN neutralizing antibodies, quantified from 5–10 images by the condition. Statistical significance calculated using the unpaired *t*-test, N = 3, was shown * *p* < 0.05).

In the light of the previous data, we decided to investigate the IFN role in HMPV–RSV interaction in dual infections. For this purpose, similar single and co-infection assays were performed in the presence or absence of neutralizing antibodies to block type I and type III IFNs. Five days post-infection, viral replication was determined by quantifying viral RNA using RT-qPCR and the percentage of infected cells from immunostaining images acquired by confocal microscopy (Figure 6). While only a slight and non-significant effect was observed in HMPV single infections, its inhibition by RSV in co-infections was significantly attenuated when type I and type III IFNs were neutralized (Figure 6A,B). In contrast to in vitro infection of A549-derived cells (Figure 5B), a surprisingly modest increase of RSV replication was observed when IFNs were blocked using neutralizing antibodies against IFN (Figure 6C,D). Although significant, this increase was not high (less than 1 and 0.5log in RNA and IF quantifications, respectively) and could be explained by a slight RSV sensitivity to type I IFN in 3D tissues (Figure 4).

Finally, processing confocal images (like in Figure 6E) from the co-infection assays allowed the determination of the percentage of co-infected cells (cells containing both RSV and HMPV). The percentage of co-infected cells was low in all conditions and never exceeded 10% (Figure 6F). Interestingly, this percentage was significantly higher in the presence of anti-type I and III IFNs. This suggests that anti-viral state inhibition by the anti-IFNs antibodies would block the interference exerted by RSV on HMPV and likely allowed their co-replication in the same host cell. In summary, in the absence of the main epithelial IFN response, the HMPV–RSV interaction was disrupted, leading to less HMPV replication inhibition and the increase of co-infected epithelial cells. These findings support the hypothesis of at least the partial IFN involvement in HMPV blockade by RSV.

## 4. Discussion

This paper is one of the first ex vivo studies of HMPV single and dual infections. It constitutes a detailed characterization of pneumoviruses’ infections of the upper respiratory tract in order to unveil mechanisms implicated in their pathogenesis, which still needs to be investigated. Limitations of the in vivo (mainly linked to animal experimentation and species specificity) and in vitro (especially morphological and functional variations in non-differentiated versus differentiated cells, respectively) models were overcome thanks to the use of three-dimensional in vitro tissues reconstituted from primary cells. Such ex vivo models are morphologically and functionally close to the airway epithelium and constitute the most suitable approach to study human viral respiratory infections [21,22]. During the 21st century, more 3D culture models, such as rotating wall vessel (RWV) and NASA 3D systems, have also been extensively used as an appropriate surrogate model to recapitulate the 3D architecture and the multicellular complexity of tissues observed in vivo and study virus-host interaction including RSV [23,24,25,26,27,28]. These models show common physiological characteristics with the human airway respiratory epithelium. They are polarized and composed of multiple differentiated epithelial cells stratified in *in-vivo*-like cellular organization. Their permeability and integrity are insured by the physiological distribution of tight junctions and they are capable of producing mucus and secrete cytokines and other immune molecules as a first line of defense against pathogens [23,24,25,26,27,28].

Theoretically, air–liquid interface culture models would reproduce better infection by air-borne pathogens of airway epithelium in patients where the apical surface is in contact with the air. Nonetheless, previous work showed that infections of RWV models faithfully reproduced in vivo infection [26]. They are also more suitable for large scale cultures and studies to analyze cellular and molecular host-pathogen responses. All these systems, using cells in ALI culture and in suspension, allow the understanding of the epithelium involvement, *per se*, in host-virus interaction during only the early steps of the infection, before the activation of immune cells and the adaptive immunity, which constitutes their main limitation.

Although comparable clinical features are frequently reported in adults and children infected by these two pneumoviruses (reviewed in [4,5]), RSV is responsible for a greater number of hospitalization in pediatric yards than HMPV and causes a higher number of bronchiolitis and pneumonia in newborns, which suggests that RSV is more involved in severe respiratory disease compared to HMPV. In this paper, comparison of their infections ex vivo strongly suggested that HMPV is less pathogenic than RSV in tissues infected with the same MOI. This was first assessed in single infections showing that HMPV replicates less efficiently and induces less host response compared to RSV (Figure 1 and Figure 2). The moderate pathogenesis induced by the recombinant RSV-A strain used in the present study was comparable with RSV-B and -A clinical strains previously studied [14,15]. HMPV behaves rather as viruses with low pathogenicity [15] in this model. Of note, in comparison to RSV infection in our model, lower levels of cytokine induction and later peak of infection have been observed in NASA 3D system using different RSV strain and MOI [26]. In line with our observations, a more recent comparative study of RSV and HMPV replication in similar ALI culture but using higher MOI (0.3 and 3 respectively) confirmed that RSV replicated better ex vivo and that both viruses infected apically without evidence of syncytia occurrence [29].

Further investigation of viral pathogenesis in dual infections showed that RSV is capable of competing against HMPV but not vice versa (Figure 3). A number of epidemiological studies have previously reported unclear or controversial conclusions regarding the type of association between RSV and HMPV [7,8,9]. Moreover, interference between RSV and HMPV viruses is barely described, as only severe cases are reported. The mechanism of RSV–HMPV interaction is still poorly studied. Previous works recently showed that respiratory virus–virus negative interaction could be mediated by the interplay between viruses and immune response and particularly IFN induction [15,18,30]. In the present work, we also provided new evidence probing the role of the epithelial anti-viral immune response in the differential pathogenesis of HMPV and RSV. Unlike RSV, HMPV was more sensitive to IFN treatment (Figure 4 and Figure 5). More interestingly, when type I and type III interferons were neutralized in co-infections, the loss of susceptibility to HMPV in RSV infected tissues was partially prevented and the proportion of co-infected cells was enhanced (Figure 6). HMPV replication would be, therefore, inhibited by its own IFN induction in single infection, while its more pronounced inhibition in dual infections would be due to the additional higher IFN stimulation by RSV. Infections performed in the presence anti-IFNs antibodies showed an unexpected non-significant increase of HMPV replication in a single infection and only partial rescue of HMPV inhibition by RSV (Figure 6A,B). This suggests that other actors from the innate immunity response (like anti-IFNλ-2 and -3 and other cytokines) would be also involved in HMPV inhibition, and that are possibly STAT1 pathway-dependent. Of note, we did not test for type II IFN as it is not expressed in the airway epithelial tissues.

Some viral proteins are used by RSV (non-structural proteins NS1 and NS2) and HMPV (small hydrophobic protein SH) as immune response modulators and might be responsible of the differential sensitivity to IFN [18,31]. The implication of their efficiency to hijack the IFN pathways in virus-virus interaction still needs to be clarified. In addition to interferon stimulation, more strategies could be used by RSV and HMPV to increase their virus spread efficiency, like inclusion body formation [32] previously reported for RSV and HMPV [29,33,34] and prevent anti-viral host response like granule stress, as previously described for RSV (reviewed in [35]). Involvement of these mechanisms in the differential pathogenicity of HMPV and RSV and/or in the resistance of RSV-infected tissues to HMPV still needs to be explored. In this work, only the role of upper respiratory epithelia response, per se, was studied. Other actors, including molecules specifically expressed by lower respiratory tracts and/or involving immune cells, could also be linked to disease exacerbation like bronchiolitis in vivo. Finally, compared to ex vivo assays, in vitro single infections using A549-derived cells could confirm the sensitivity of HMPV to IFN but did not show any increase of RSV replication when IFN pathways were blocked. Because of the fragility of co-infected A549-derived cells, attempts of dual infection assays in 2D cultures were not successful, which highlights again the limitations of this model and the pertinence of the ex vivo model used to study respiratory viral infections.

In conclusion, this study extended our knowledge about pneumoviral infections in a highly relevant experimental model. It highlights the differential pathogenesis of RSV and HMPV and suggests the involvement of the epithelial innate immune response in virus-host and virus-virus interactions in single and dual infections. This could eventually be of interest to improve the care of young and at-risk patients in the hospital settings and to support development of appropriate treatments or preventive tools against pneumoviruses.

## Data Availability

No new data were created or analyzed in this study. Data sharing is not applicable to this article.

## Supplementary Materials

The following are available online at https://www.mdpi.com/1999-4915/13/1/139/s1, Figure S1: 3D views of immunofluorescence images of RSV- and HMPV-infected tissues. Images acquired by confocal microscopy (6 sections/tissue) from RSVmcherry (red cells, left panel) and HMPV/GFP (green cells, right panel) infected tissues after fixation and nuclei (blue) staining at 5 dpi were processed by Imaris V 9.6 software for 3D reconstruction. Of note, in Figure 1C, only the upper section from the same set of images of each infected tissue has been shown. In order to show virus localization, 3 different views were presented for each infected tissue. Figure S2: Control of IFN pathways knock out in A549-derived cells. IFN-λ or-α pretreated or untreated A549 CTRL, STAT1 KO, and IL-28RA KO cells were infected by HMPV (**A**) and RSV (**B**), MOI around 0.04. The percentage of HMPV and RSV infected cells was represented relative to untreated A549 CTRL. Statistical significance was calculated using two-way ANOVA, N = 2. Video S1: Cilia beating in mock-infected tissue at 5 dpi. Movies were obtained using contrast phase microscope and image ProsPlus V2.0 software. Video S2: Cilia beating in RSV-infected tissue at 5 dpi. Movies were obtained using contrast phase microscope and image ProsPlus V2.0 software. Video S3: Cilia beating in HMPV-infected tissue at 5 dpi. Movies were obtained using contrast phase microscope and image ProsPlus V2.0 software.
