# Supplementary figures and images for: RSV and HMPV Infections in 3D Tissue Cultures: Mechanisms Involved in Virus-Host and Virus-Virus Interactions
Source: Viruses. 2021 Jan 19;13(1):139. doi: 10.3390/v13010139 (PMC7835908; doi:10.3390/v13010139)

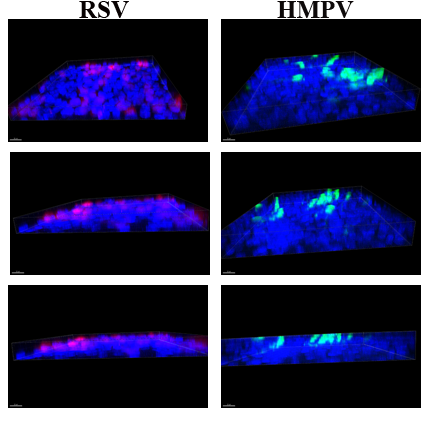

Supplement: Supplementary file 1 [file viruses-13-00139-s001.zip › Geiser et al 2020 supp data/Figure S1.tif]

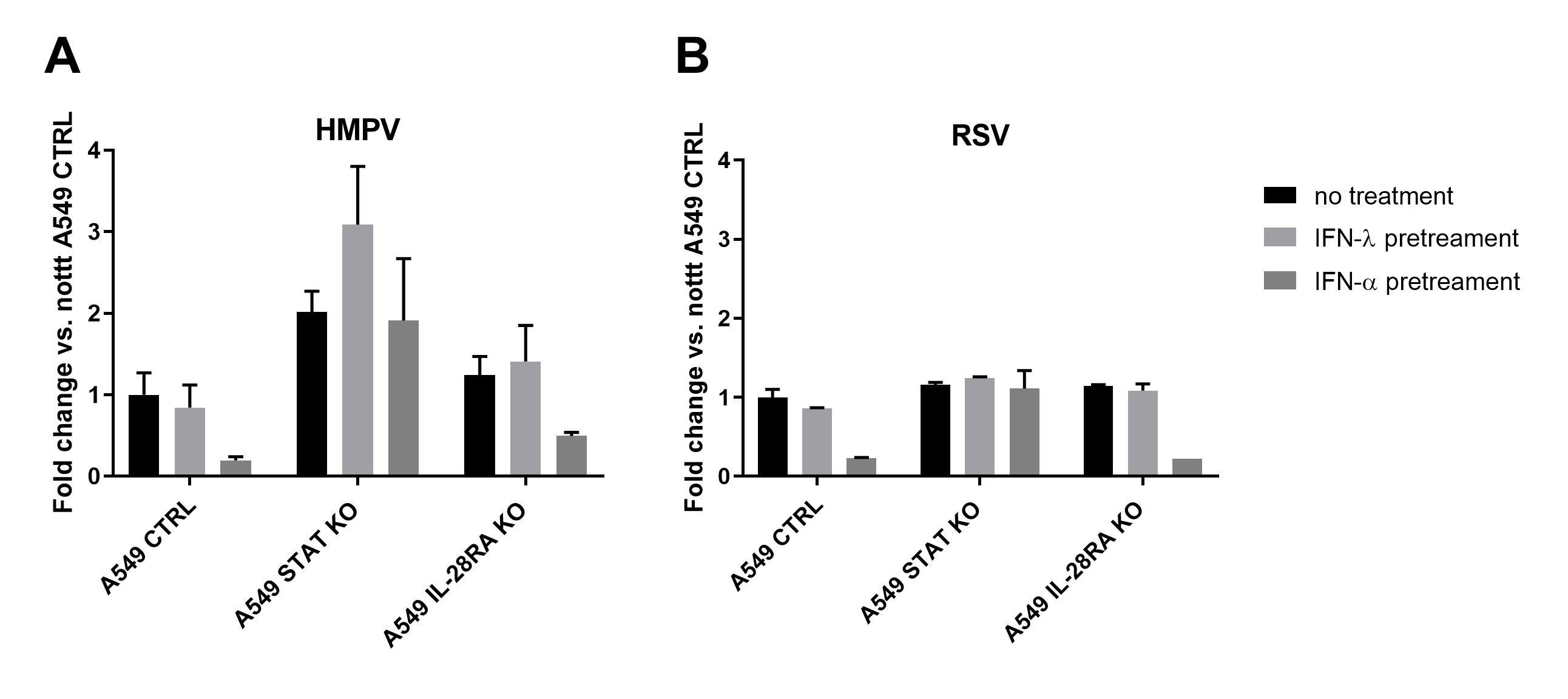

Supplement: Supplementary file 1 [file viruses-13-00139-s001.zip › Geiser et al 2020 supp data/Figure S2.tif]
